# The identification of *H3F3A* mutation in giant cell tumour of the clivus and the histological diagnostic algorithm of other clival lesions permit the differential diagnosis in this location

**DOI:** 10.1186/s12885-018-4291-z

**Published:** 2018-04-02

**Authors:** Federica Scotto di Carlo, Giuseppina Divisato, Maurizio Iacoangeli, Teresa Esposito, Fernando Gianfrancesco

**Affiliations:** 1 Institute of Genetics and Biophysics “Adriano Buzzati-Traverso”, National Research Council of Italy, Naples, Italy; 2 Department of Environmental, Biological and Pharmaceutical Sciences and Technologies (DiSTABiF), University of Campania “Luigi Vanvitelli”, Caserta, Italy; 3 Department of Neurosurgery, Umberto I General Hospital, Ancona, Italy; 4 IRCCS INM Neuromed, Pozzilli, Italy

**Keywords:** Giant cell tumour, Clivus, *H3F3A* gene, Diagnostic algorithm, Differential diagnosis

## Abstract

**Background:**

Giant Cell Tumour of Bone (GCT) is a locally aggressive primary bone tumour that usually occurs at the epiphyses of the long bones of the appendicular skeleton with a tendency to recurrence. Recurrent somatic *H3F3A* mutations have been described in 92% of GCT cases. GCTs involving the Clivus are extremely rare lesions and less than 15 cases are described in the literature. They represent a surgery challenge and are easily misdiagnosed. Our aim was to reveal if the genetic bases underlying Clival GCTs were the same of GCTs of long bones to improve the diagnosis and treatment.

**Methods:**

The targeted somatic sequencing of GCT-related genes (*H3F3A*, *H3F3B*, *IDH1*, *IDH2* and *ZNF687*) was performed on Clival GCT biopsies of two different cases. Histological analyses on the same tissues were used to detect the neoplastic population and its expression profile.

**Results:**

Sanger sequencing revealed that both patients were positive for the p.Gly34Trp mutation in the *H3F3A* gene. Immunofluorescence assay using monoclonal antibody, specifically detecting the mutant H3.3, highlighted that the mutation only involved the mononuclear cell population and not the multinucleated giant cells. Moreover, immunohistochemistry assay showed that RANKL was highly expressed by the stromal cells within Clival GCT, mimicking what happens in GCT of the long bones. In addition, systematic literature review allowed us to generate a histology-based diagnostic algorithm of the most common clival lesions.

**Conclusions:**

We conclude that the Clival GCT is genetically defined by somatic mutation in the *H3F3A* gene, linking it to the GCT of long bones. The similarity with GCTs of long bones let us to hypothesize the utility of Denosumab therapy (already effective for GCTs) in these surgically challenging cases. Moreover, *H3F3A* genetic screening can be combined to the histological analysis to differentiate GCTs from morphologically similar giant cell-rich sarcomas, while the histological diagnostic algorithm could help the differential diagnosis of other clival lesions.

## Background

Giant cell tumour of bone (GCT) is a primary intramedullary neoplasm that accounts for 5% of skeletal tumours, composed by numerous multinucleated osteoclast-like giant cells evenly scattered throughout the mass and ovoid or spindle mononuclear stromal cells [1]. GCT is generally considered a benign tumour, even though it is characterised by localised bone destruction due to the osteolytic properties of osteoclast-like giant cells that express the markers involved in bone resorption activity [2]. Although the giant cells are a significant part of this tumour, the stromal cells constitute the actual neoplastic component. Indeed, Behjati et al. recently described recurrent driver somatic mutations in the *H3F3A* gene only restricted to the stromal cell population, rather than to cells of the osteoclast lineage, demonstrating that GCT is a mesenchymal neoplasm [3]. Particularly, an exquisite specificity for *H3.3* alterations was observed among different bone tumours, emphasizing the importance of genotyping tumours for diagnostic purposes [3–5]. On the contrary, we recently highlighted that giant cell tumour, when arising on Paget’s disease of bone – a disorder of bone remodelling – shows a different genetic signature characterised by a germline mutation in the *ZNF687* gene [6, 7].

Even though giant cell tumour shows a low potential for metastasis, it may locally recur at high rate [8]. Therefore, a complete resection accompanied by adjuvant therapy to prevent tumour recurrence is required. Denosumab has been demonstrated as an effective therapy in GCT for tumour control. This fully humanised monoclonal antibody selectively targets RANKL, thus inhibiting its interaction with RANK receptor on the surface of osteoclast precursors and preventing bone destruction activity [9]. Denosumab treatment in GCT has been shown to effectively reduce not only the number of giant cells but also the relative content of proliferative stromal cells, promoting new bone formation [9, 10].

GCT typically occurs when the growth plate has closed and therefore is frequently observed in skeletally mature individuals with its peak incidence in the third and fourth decade of life [11]. The majority of GCTs develop as single lesions and are located at the epiphyses of long bones, predominantly affecting the distal femur, the proximal tibia, the distal radius and the proximal humerus [12, 13]. GCTs involving other anatomic sites are uncommon and only less than 1% of all reported GCTs occurs in the skull, where they preferentially affect the sphenoid and temporal bone [14]. Specifically, primary giant cell tumours of the clivus are extremely rare lesions, with less than 15 cases described in the literature, that typically present with compression of the cranial nerves and consequent diplopia, headache and deafness [15–26]. Albeit histologically benign, Clival GCT can be clinically devastating because of its anatomical location and destruction of vital structures [15]. The tumour also shows a high tendency to local recurrence, thus making the total surgical resection essential [15, 19]. However, the complete removal is not always feasible and an adjuvant treatment (chemotherapy or radiotherapy) is often used [15–17, 20, 24].

In the present article, we defined the genetic basis of giant cell tumour of the clivus, demonstrating the presence of somatic mutation in the *H3F3A* gene in tumour biopsies of two patients, highlighting that Clival GCT can be considered like GCT of long bones.

## Methods

### Patients and tissues

The patient material comprises primary giant cell tumours of the clivus. Tissue samples were obtained as Formalin Fixed Paraffin Embedded (FFPE) specimens from 2 patients surgically treated in Università Vita-Salute San Raffaele, Milan, Italy (patient 1) and Department of Neurosurgery, Umberto I General Hospital, Ancona, Italy (patient 2).

### DNA extraction

Sections (7-μm-thick) were cut from FFPE tissue blocks and subjected to DNA extraction with GeneRead DNA FFPE Kit (Qiagen), following the manufacturer’s instructions.

### Genetic screening

Mutation analysis of GCT-related genes (*H3F3A, H3F3B, IDH1, IDH2*, and *ZNF687*) was conducted by PCR followed by Sanger sequencing, as recently reported [7].

### Allele-specific sequencing

The molecular cloning of *H3F3A* DNA sequence containing c.G103T mutation was carried out as previously described [7]. Briefly, *H3F3A* genomic region was amplified as described above, subcloned into pJET1.2/blunt cloning vector (Thermo Fisher Scientific), and subjected to Sanger sequencing.

### Immunohistochemistry and immunofluorescence analysis

Tumour sections (7-μm-thick) of paraffin blocks were cut using a microtome and collected on Superfrost glass slides (Thermo Fisher Scientific). FFPE tumour tissues were then deparaffinised and rehydrated as we recently reported [7]. Sections were incubated with primary antibodies for rabbit monoclonal anti-H3.3 p.Gly34Trp (RevMab Biosciences, clone RM263, recently used by Lüke et al.), mouse monoclonal anti-TRAP (Thermo Fisher Scientific MA5–12387), mouse monoclonal anti-Tenascin C (Abcam ab6393) and rabbit polyclonal anti-RANKL (Abcam ab9957) [27]. Immunofluorescence sections were observed using a Nikon’s A1R confocal laser microscope, while immunohistochemistry sections were analysed using Nikon Intensilight C-HGFI.

## Results

### Clinical case description

**Fig. 1:**
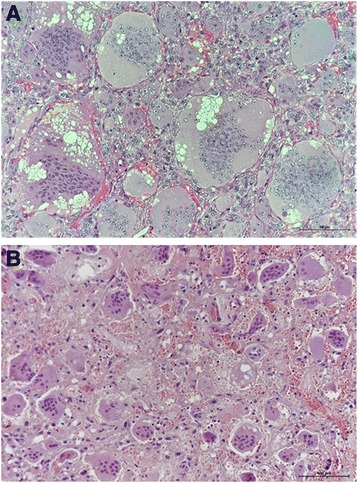
Case presentation. **a** Histology of the specimen excised in 2014 from patient 1, showing numerous multinucleated osteoclast-like giant cells scattered in a background of mononuclear cells (hematoxylin and eosin stain; 20× objective). **b** Haematoxylin and eosin staining of tumor biopsy of patient two. Smaller osteoclast-like giant cells are observable (20× objective)

**Fig. 2:**
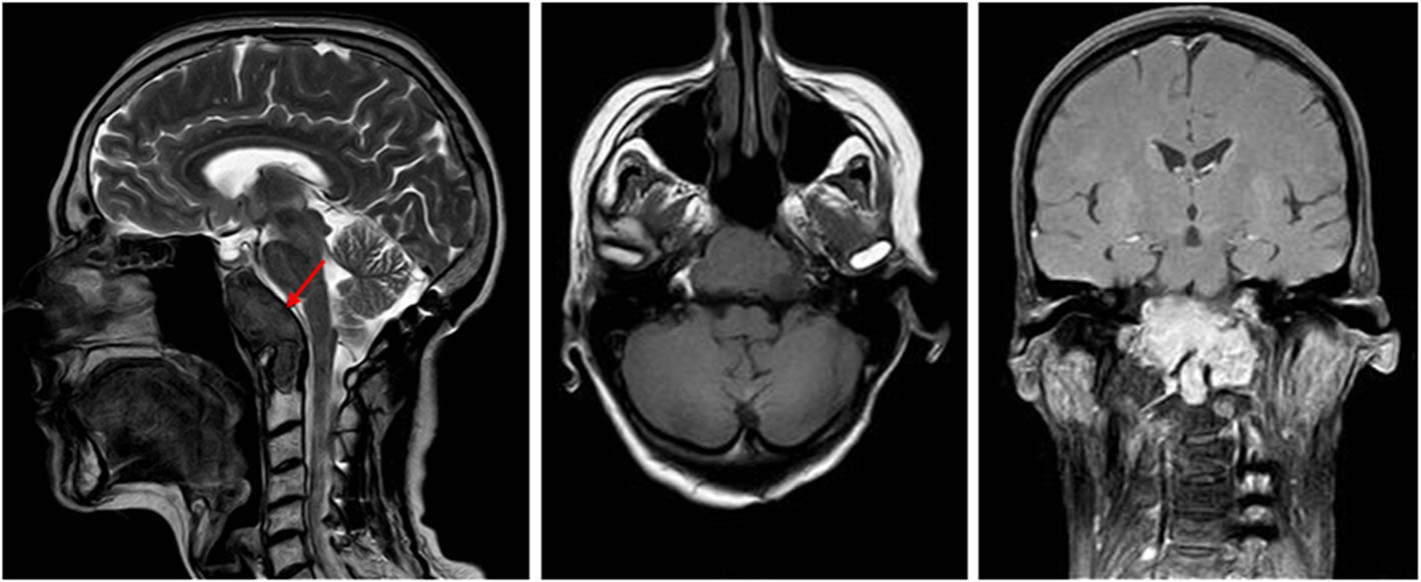
MRI images. Sagittal, axial and coronal magnetic resonance images of patient 1, depicting the large and recurrent giant cell tumour originating from the clivus (indicated by the red arrow in the sagittal view), with a C1 vertebral infiltration

We recently recruited a 55-year-old female (hereafter referred to as “patient 1”), diagnosed in 2014 with giant cell tumour of the clivus. Since 2013, she complained holocranial headache associated with vomiting, tongue numbness and difficulty with swallowing and speech. Computed tomography (CT) showed a lytic mass 5 cm in size involving the clivus. Magnetic resonance imaging (MRI) revealed a lobulated mass (5 × 2,8 × 3,8 cm) originating from the clivus and extending into the sella and the epistropheus. In 2014, she underwent surgical removal of the mass through suboccipital approach and from the postoperative histopathology the diagnosis of Clival GCT was made. The tumour was highly vascularized and composed of numerous osteoclast-like giant cells that were bigger than those usually described in GCTs, with 50 to 70 nuclei, scattered on a background of mononuclear cells (Fig. 1a). However, one month after the surgical intervention, the tumour mass grew, symptoms presented again and the mass was newly removed. Postoperatively, the patient received tomotherapy against the residual lesion but only dysphonia improved. Nevertheless, so far in 2016 she suffers of worsening headache, tongue paralysis and dysphagia. MRI demonstrated that a recurrence occurred and showed growth of the tumour (5,3 × 2,1 × 3,9 cm), with compression of hypoglossal nerve (Fig. 2). She has been planned for resurgery.

In 2013, the patient 2 was operated through endoscopic endonasal approach to remove the giant cell tumour of the clivus, responsible for his progressive headache and diplopia. After surgery, the patient was asymptomatic and did not undergo radiation therapy. Brain MRI, regularly performed up to six years after the operation, revealed that he did not present any relapse of the disease [18]. The haematoxylin and eosin staining that we performed on tumour sample confirmed the clinical diagnosis, even though the size of giant cells was remarkably smaller than in patient 1 (Fig. 1b).

### *H3F3A* somatic mutation defines the molecular basis of Clival GCT

**Fig. 3:**
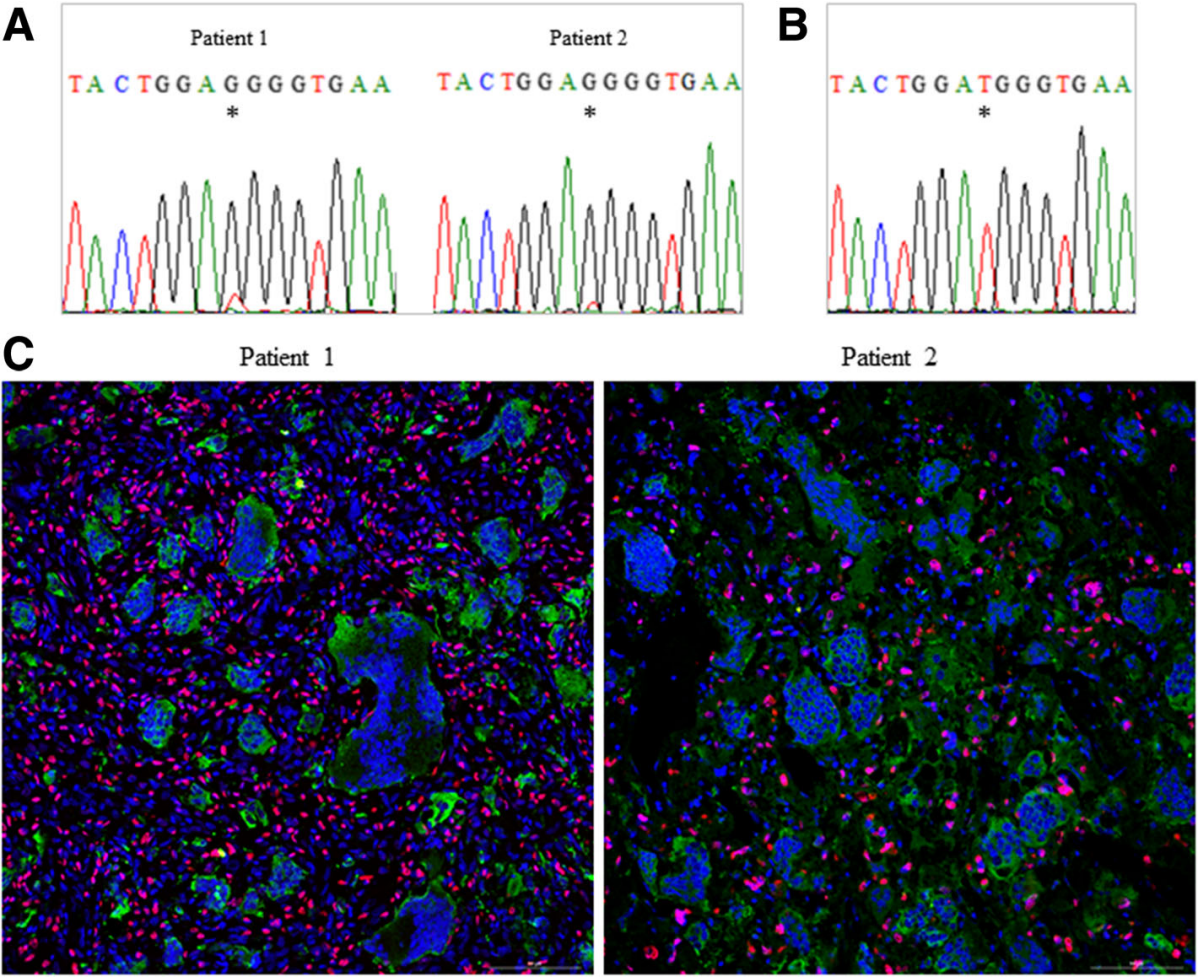
*H3F3A* mutation causes GCT of the clivus. **a** Sequence electropherograms of the PCR product of the *H3F3A* gene, showing the low mutational peak at position c.103 in patient 1 (left) and patient 2 (right). Asterisks denote the mutated base. **b** Representative electropherogram of patient 1 showing the allele specific sequencing of cloned DNA fragment and demonstrating the presence of p.Gly34Trp mutation in *H3F3A*. **c** Immunofluorescent visualization of the *H3F3A* mutation in the nuclei of mononuclear cells, while osteoclastic giant cells are negative and only react with TRAP antibody (20× objective, scale bar 100 μm). Note the higher number of neoplastic cells in Clival GCT of patient 1 (left) compared to patient 2 (right)

The molecular bases underlying Clival GCT pathogenesis have never been investigated, likely because of the extreme rarity of this skull tumour. Here, in an attempt to prove that GCT of the clivus may be caused by the same genetic alteration of GCT of long bones, we performed the molecular analysis of the GCT-related genes (*H3F3A, H3F3B, IDH1* and *IDH2*) as well as of the *ZNF687* gene that we recently identified as gene responsible for giant cell tumour associated with Paget’s disease of bone [3, 6, 28]. We analysed the tumour specimens of the two patients described above and revealed that both harboured the heterozygous p.Gly34Trp mutation in the *H3F3A* gene; whereas the coding regions of the other analysed genes resulted negative for any mutations (Fig. 3a). Moreover, we performed the genetic screening on DNA extracted from peripheral blood of patient 1 and, as expected, we did not detect the *H3F3A* alteration, thus confirming that the mutation was somatically acquired. Given the somatic origin of the mutations, Sanger sequencing detected a very low peak in correspondence of the investigated nucleotide in both patients, making the signal from the mutations hardly distinguishable from background noise. Therefore, we cloned both DNA fragments carrying the p.Gly34Trp mutation and, as shown in Fig. 3b, we confirmed the presence of the mutant allele.

### Stromal cells are the malignant component of Clival GCT and express high levels of tenascin-C

Considering that our samples of Clival GCT showed a different clinical aggressiveness of the tumour, we aimed at defining the amount of neoplastic cells within the mass through immunofluorescence assay, using a mutation-specific monoclonal antibody. We demonstrated that the p.Gly34Trp mutation in *H3F3A* disturbs the mononuclear cells that showed a strong nuclear staining in the tumour biopsies of both patients, whereas, as expected, the nuclei of all TRAP-positive giant cells were negative, indicating that the mutation only regarded the mononuclear cell compartment (Fig. 3c). This analysis allowed us to observe that the number of malignant cells was higher in the tumour biopsy of patient 1, correlating with her more severe phenotype.

**Fig. 4:**
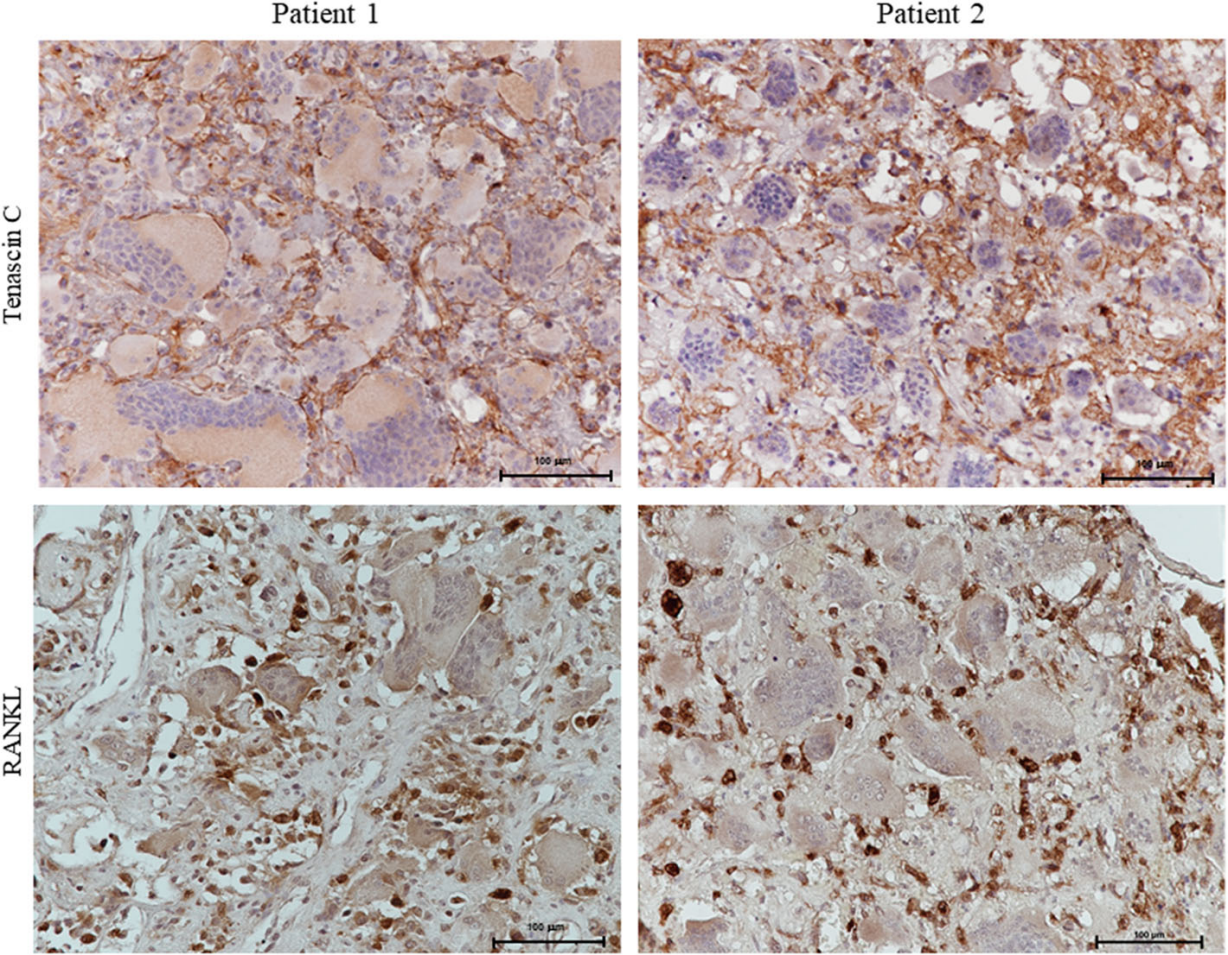
Clival GCT expresses Tenascin-C and high levels of RANKL. Tenascin-C reactivity in the extracellular matrix and RANKL-positive mononuclear cells in tumour biopsies of patient 1 (left) and patient 2 (right) are shown (20× objective, scale bar 100 μm)

Given the significant expression of the glycoprotein Tenascin-C in GCT patients and its association with local relapse, we also evaluated its immunohistochemical expression in both tumour biopsies. Interestingly, we found strong immunopositivity also in these two Clival GCTs, with a reticulate organization pattern in the extracellular matrix (Fig. 4). This result is in agreement with the local recurrence of the tumor in patient 1, occurred two years after the surgical operation.

### Clival GCTs express high levels of RANK-ligand mimicking GCT of long bones

It is well known that GCTs of long bones express high levels of RANKL, justifying the introduction of the monoclonal antibody Denosumab in GCT clinical practice [9]. To reveal if Clival GCT and GCT of long bones shared a similar expression pattern, we also evaluated RANKL levels in the Clival GCT biopsies of both patients. Immunohistochemistry assay detected a high number of RANKL-positive cells, showing an expression pattern similar to that recently reported for GCT of long bones by us and others (Fig. 4) [7]. This result led us to speculate that Denosumab treatment could represent an effective therapy for this surgically challenging tumour.

### Systematic literature review confirms that skull GCTs are rare entities

Giant cell tumours seldom occur in the skull. Reviewing data from three extensive studies in the literature, we were able to estimate that the frequency of GCTs arising from the calvarial bones is 0,51% of all GCTs. In 1985, among a collection of 407 cases of GCT, David Dahlin highlighted only 4 tumours occurring in the skull [29]. Subsequently, Bertoni et al. reviewed 2046 GCT cases (546 of which were contained in the Mayo Clinic files) and found only 15 cases affecting the skull bones [14]. In the Rizzoli Case Archive, among 1449 GCT cases collected from 1900 to 2012, only 1 was located at skull [30]. Therefore, skull GCTs have been observed only in 20 out of 3902 GCT patients.

**Fig. 5:**
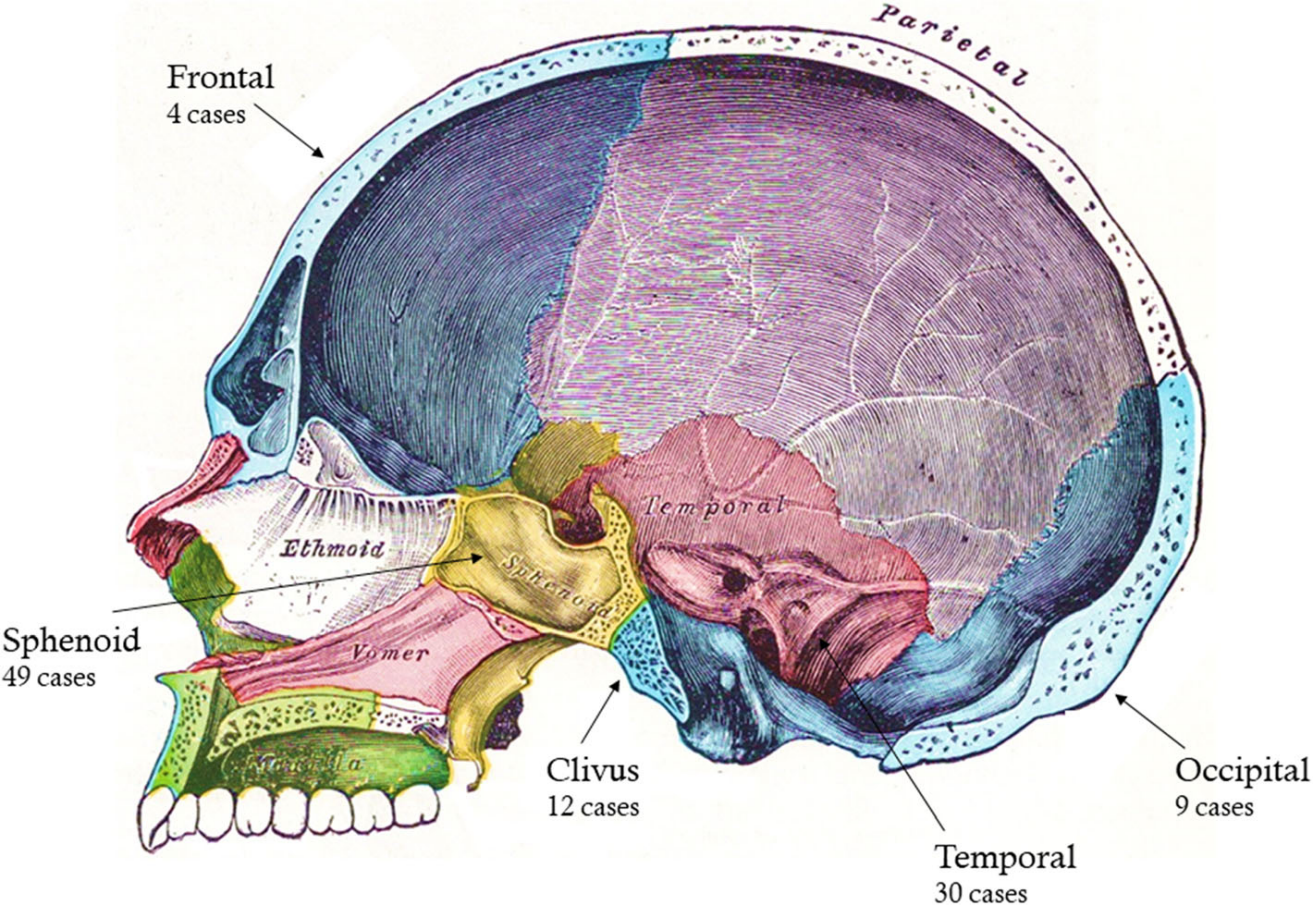
Diagrammatic representation of the human skull in sagittal section, identifying the main bones and cavity. The location of lesions in 104 cases of skull GCTs is indicated by arrows. Adapted from *Anatomy of the Human Body* [77]

Moreover, we analysed 104 case reports of GCTs of the skull base from 1969 to 2017 and highlighted that the sphenoid and temporal bones were those preferentially involved by GCT, with a frequency of 47% and 28%, respectively [14, 29, 31–45]. Clivus as primary site of GCT was described in 12 out of 104 reports (12%), while occipital and frontal GCTs showed an incidence of 9% and 4%, respectively (Fig. 5) [15–26, 38, 46–51].

### Histological diagnostic algorithm permits the differential diagnosis of the main clival lesions

Giant cell tumour is among the rarest lesions arising within the clivus but pathologies of the clivus are represented by a wide range of diseases, and hence the differential diagnosis can include: chordoma; chondrosarcoma; meningioma; osteosarcoma; pituitary adenoma; lymphoma; and plasmacytoma. These tumours have similar anatomical characteristics and imaging approaches are not sufficient to distinguish them. However, differentiating among these masses is essential to define the proper pharmacological treatment. Here, we generated a diagnostic algorithm for the most frequent lesions, showing in Table 1 their gross and microscopic appearance as well as their distinctive immunostaining pattern.

**Table 1:** Histology-based diagnostic algorithm of the main lesions arising within the clivus

	*Histological appearance*		*Immunostaining pattern*							
	Gross examination	Microscopic appearance	S100	EMA	Keratin	Vimentin	Brachyury	CD3	CD20	CD45
Chordoma	Soft, haemorrhagic and grey lobulated tumour	Large cells with vacuolated cytoplasm	Yes	Yes	Yes	Yes	Yes	No	No^b^	No^b^
Chondrosarcoma	Hard, grey-white lobulated and cartilaginous mass	Large bubbly-looking cells with stellate-shaped nuclei	Yes	No	No	Yes	No	No	n/a	n/a
Meningioma	Round and well circumscribed mass attached to the dura	Syncytial cells with eosinophilic cytoplasm and vesicular nuclei	Yes	Yes	Yes^a^	Yes	No	No	n/a	No^b^
Osteosarcoma	Bulky, grey-white mass with areas of haemorrhage	Spindle cells producing eosinophilic matrix (osteoid). 25% show osteoclast-like giant cells	Yes	No	No	Yes	No	No	No^b^	No^b^
Pituitary adenoma	Soft, cream-colored tumour from the sella turcica, usually invading adjacent structures	Round cells with eosinophilic cytoplasm and spherical nuclei	No	No	Yes	Yes	No	No	No^b^	No^b^
T-cell Lymphoma	Well circumscribed tumour mass, resembling inflammatory lesions	Highly vascularized tumour with small and round cells	No	No	No	Yes	No	Yes	No	Yes
B-cell Lymphoma	Well circumscribed tumour mass, resembling inflammatory lesions	Highly vascularized tumour with small and round cells	No	No	No	Yes	No	No	Yes	Yes
Plasmacytoma	Soft and lobulated tissue, slightly pink- or cream-colored	Sheets of mature and immature plasma cells, with eccentric nuclei and “clock-face” chromatin	No	Yes	Yes	Yes	n/a	No	No	No

^a^Seventy-five percent of malignant meningioma express cytokeratin; while no expression was found in benign meningiomas [60]

^b^The negative staining refers to neoplastic cells. Immune infiltrating cells can be responsible for a positive staining

Chordomas are the most common pathologies in the skull, accounting for 42% of all chordomas, and usually occur in the vicinity of the clivus [52, 53]. They arise from notochord remnants and, although slow-growing, they are locally aggressive and tend to recur after surgical removal, behaving like malignant tumours [52]. Chondrosarcomas comprise 6% of skull base tumours and develop from the transformation of mesenchymal cells producing cartilage. They show a high predilection for the petroclival synchondrosis, and yet they can also have a midline skull base location [54, 55]. Therefore, chordomas and chondrosarcomas may occupy the same anatomic location, show the same clinical symptoms (headache and diplopia) and a similar MRI appearance (hypointensity on T1 and hyperintensity on T2), and indeed the two tumours are frequently confused with one another [56]. Nevertheless, chordomas show a substantially worse prognosis and positivity to the markers EMA, cytokeratin and brachyury helps to distinguish it from chondrosarcoma [57] (Table 1).

Meningiomas are tumours originating from the arachnoidal cells and, being the clivus covered with dura mater, it can be affected. Specifically, meningiomas more frequently arise in the petroclival junction. Patients commonly present with headache, seizures and hearing disturbance. This tumour has a dual mesenchymal and epithelial differentiation potential, and hence shows positivity to both vimentin and keratin [58, 59]. Of note, keratin expression can be observed in 75% of malignant meningiomas; whereas its expression is never detected in benign meningiomas [60] (Table 1).

Osteosarcomas are highly aggressive lesions caused by osteoid-producing malignant cells. They primarily affect the metaphysis of long bones but 6–10% of them may take place within the skull [61]. Being a tumour of mesenchymal origin, osteosarcoma shows positivity to vimentin. However, the presence of osteoid as well as the positivity to alkaline phosphatase represent the criteria for the differential diagnosis of osteosarcomas [62].

Pituitary adenomas are common intracranial tumours, comprising about 10% of skull lesions. They frequently invade the adjacent structures, even though clival involvement is rarer. These lesions can be asymptomatic or can cause headache and amenorrhea [63].

Lymphomas and plasmacytomas are cancers of the immune system; lymphomas develop from lymphocytes, while plasmacytomas are monoclonal proliferation of plasma cells. The sites most commonly affected are bones with active bone marrow hematopoiesis and the skull is seldom involved [64, 65]. Their microscopic appearance is very similar but negative staining for markers such as CD45 and CD20 is useful in supporting the plasmacytoma as differential diagnosis [66] (Table 1).

On the other hand, clivus can also be the site of metastatic lesions, commonly arising from prostate carcinoma (18,1%), hepatocellular carcinoma (10,6%), and thyroid follicular carcinoma (8,5%). Although they are more uncommon than primary clival tumours, they should be considered in the differential diagnosis that should be made using the histological markers specific for the primary cancers [67–69].

## Discussion

Giant cell tumours of the skull account for 0,51% of all GCTs and tend to affect the sphenoid and the temporal bone. The selective and preferential occurrence of the tumour in these bones rather than other calvarial bones may depend on their embryologic origin: as in the case of long bones, also the sphenoid and the temporal bone are generated through endochondral bone formation; whereas the other skull bones are produced by an intramembranous formation [14]. In fact, we pointed out that the occipital and the frontal bones are the cranial sites less frequently involved by GCT. The giant cell tumour of the clivus is also a very rare lesion, with only 12 cases described to date [15–26]. However, the incidence of the Clival GCT (12% of all skull GCTs) is not the lowest of all and this may be due to its location at the sphenooccipital synchondrosis, a joint composed of regions of endochondral ossification. The clinical aggressiveness of Clival GCT is strongly related to its complicated anatomical location, which implies multiple cranial nerve involvement with subsequent headache, decreased vision, visual field defect, diplopia, ophthalmoplegia, and deafness [18, 20]. The traditional approaches for diagnosis of GCT of the clivus comprise skull X-ray, computed tomography and magnetic resonance imaging but imaging studies alone are not enough to distinguish Clival GCT from other bone lesions. Indeed, the definitive diagnosis is only postoperative, based on biopsy findings and the main histomorphologic feature is the presence of giant cells similar to osteoclasts [20]. However, multinucleated osteoclast-like giant cells can be found in several malignant and nonmalignant bone lesions (i.e. osteosarcomas, chondroblastomas, giant cell granulomas, giant cell reparative granulomas, and brown tumours of hyperparathyroidism); therefore, the postoperative histopathology can be ambiguous and Clival GCT can be easily misdiagnosed [16, 20, 24].

In this study, we unveiled the genetic basis of the giant cell tumour of the clivus, analysing the tumour specimens of two patients affected by this aggressive bone lesion. We analysed the coding regions of *H3F3A*, gene mutated in most cases of giant cell tumour of long bones. Indeed, since 2013, *H3.3* mutations have been identified in more than 90% of all GCT cases analysed, with the p.Gly34Trp as the most frequently harboured mutation [4, 5, 70–72]. We also examined the mutational status of *H3F3B, IDH1* and *IDH2* that, though more rarely, have been associated to GCT [3, 5, 28]. Besides, we investigated the presence of mutation in the coding region of the *ZNF687* gene that we recently identified in giant cell tumour complicating Paget’s disease of bone [6]. The latter is a localised disorder of excessive and abnormal bone remodelling that can also affect the skull base, especially the clivus and surrounding sphenoid bone structures [73]. Considering this frequent localisation (42%) as well as the evidence that the tumour biopsy of patient 1 was composed of giant cells whose size resembled that found in pagetic GCT, we sought to investigate whether these two clival masses could represent GCT degeneration of pagetic lesions [7]. Interestingly, we highlighted that both tumour samples harbour the p.Gly34Trp mutation in the *H3F3A* gene and demonstrated that the change was somatically acquired, as it was not found in the peripheral blood. Moreover, our immunofluorescence analysis further confirmed the restriction of the *H3F3A* mutation to the stromal cell population that was abundant in both tumour biopsies. Consequently, even though in a relatively low number of samples, we demonstrated that two cases of GCT affecting the clivus are genetically defined by mutation in the *H3F3A* gene and hence, Clival GCT can be considered a typical giant cell tumour with a peculiar anatomical localisation. Therefore, the mutational analysis of the *H3F3A* gene is an accurate tool for the diagnosis of Clival GCT that can be combined with the histological analysis of the tumour sample in order to distinguish the giant cell tumour from other osteoclast-rich tumours. In agreement, it has been recently demonstrated that *H3F3A* mutations were not detectable in neither giant cell-rich sarcomas nor giant cell-rich benign lesions, whereas almost all GCT tissues carried *H3F3A* mutation [4, 71]. While the *H3F3A* genetic screening allows to distinguish GCT from other giant cell-rich lesions, the histology-based diagnostic algorithm that we generated in this study may be useful to differentiate other morphologically overlapping lesions arising within the clivus.

We also found a strong Tenascin-C immunoreactivity in the two Clival GCT biopsies, indicative of an aggressive tumour with a tendency to recur in situ, as this extracellular glycoprotein is highly expressed in the microenvironment of most solid tumours to promote migration and epithelial-mesenchymal transition [74]. As a matter of fact, the patient 1 have had a local recurrence of the tumour and has been recently planned for resurgery. However, Tenascin-C immunoreactivity can also be found in central giant cell granulomas, making it not a suitable histological marker to distinguish GCTs from other giant cell-rich lesions [75]. Nevertheless, the expression of Tenascin-C in both tumour samples further links the Clival GCT to the GCT of long bones, where its intense expression in the extracellular matrix has been clearly described by us and others [7, 74, 75]. Finally, the ability of mononuclear cells within Clival GCT to produce high levels of RANKL, mimicking what happens in GCT of long bones, led us to speculate that Denosumab treatment could represent an effective therapy for Clival GCT.

Overall, these results demonstrate that Clival GCTs are caused by the same genetic defect of GCTs of long bones and allowed us to hypothesize that the two tumour types can be managed using the same approaches. The gold standard for management of this tumour contemplates the surgical resection, followed by adjuvant treatment with chemotherapy or radiotherapy [15–17, 20, 24]. However, no effective chemotherapeutic agents have yet been identified and some patients developed osteosarcoma transformation at sites of previous irradiation [15, 23, 76]. To date, only one case of Clival GCT has been treated with Denosumab after surgical resection by endoscopic endonasal transsphenoidal surgery and the treatment proved to be effective in preventing tumour growth [23].

## Conclusions

We conclude that the Clival GCT is genetically defined by somatic mutation in the *H3F3A* gene. Our data also suggest that the GCT of the clivus can be treated on the same principles as that of GCT of long bones and that the relatively more aggressive phenotype of Clival GCTs should only be addressed to their anatomical location.

## Abbreviations

CT: Computed tomography
FFPE: Formalin Fixed Paraffin Embedded
GCT: Giant cell tumour
*H3F3A*: Human gene encoding H3 Histone Family Member 3A
*H3F3B*: Human gene encoding H3 Histone Family Member 3B
*IDH1*: Human gene encoding Isocitrate dehydrogenase 1
*IDH2*: Human gene encoding Isocitrate dehydrogenase 2
MRI: Magnetic resonance imaging
RANK: Receptor Activator of Nuclear Factor κ B
RANKL: Receptor activator of nuclear factor kappa-B ligand
TRAP: Tartrate-resistant acid phosphatase
*ZNF687*: Human gene encoding zinc finger protein 687
